# Lake Trout (*Salvelinus namaycush*) Naturally Infected with *Salmovirus salmonidallo3* (SalHV-3; Family *Alloherpesviridae*) Continue to Harbor the Virus for Nearly a Decade

**DOI:** 10.3390/v17111466

**Published:** 2025-10-31

**Authors:** Megan A. Shavalier, Mohamed Faisal, Thomas P. Loch

**Affiliations:** 1 Aquatic Animal Health Laboratory, Aquatic Animal Disease Ecology Program, Michigan State University, East Lansing, MI 48824, USA; shavali1@msu.edu (M.A.S.); faisal@msu.edu (M.F.); 2 Department of Fisheries and Wildlife, College of Agriculture and Natural Resources, Michigan State University, East Lansing, MI 48824, USA; 3 Department of Pathobiology and Diagnostic Investigation, College of Veterinary Medicine, Michigan State University, East Lansing, MI 48824, USA

**Keywords:** herpesvirus, lake trout, Great Lakes, fish disease, fishery conservation, latency, virus pathogenesis, *Alloherpesviridae*

## Abstract

*Salmovirus salmonidallo3* (SalHV-3) causes Epizootic Epitheliotropic Disease (EED), which has resulted in the death of millions of lake trout (*Salvelinus namaycush*) over the past 40 years. Although advancements in understanding this virus’s pathogenicity and control strategies have been made, the duration and effects of chronic SalHV-3 infections remain unknown. This study focused on lake trout that survived a natural outbreak of EED in 2012 and were maintained under quarantine conditions until 2021. Following exposure to either repeated or intermittent handling stress designed to mimic typical hatchery practices, SalHV-3 was detected (via a SalHV-3-specific quantitative PCR assay) in multiple tissues from multiple fish. Non-lethally collected tissues revealed the highest prevalence and virus loads in the fin and mucus. SalHV-3 was detected in these tissues throughout the study period (49 days, 8 sampling events), with some fish having detectible virus on each study day and others only intermittently (*n* = 1–7 sampling days). Tissues collected lethally yielded SalHV-3 detections in multiple nervous tissues, as well as in the cornea of several fish. Experiments to evaluate virus shedding revealed that SalHV-3 was intermittently detectable in fish holding water. Collectively, results indicate that lake trout can remain SalHV-3 infected for nearly a decade and intermittently shed the virus, constituting a threat to hatchery-based lake trout conservation efforts in the Great Lakes basin.

## 1. Introduction

*Salmovirus salmonidallo3* (SalHV-3), the causative agent of Epizootic Epitheliotropic Disease (EED), is a large, double-stranded DNA virus in the family *Alloherpesviridae* (Order *Herpesvirales*) [1] that has led to millions of mortalities in hatchery-reared lake trout (*Salvelinus namaycush*) since its discovery in the 1980s [2,3,4]. Disease signs associated with EED (as reported during resurgence events in 2012 and 2017) include ocular hemorrhage, corneal opacity, gill pallor, skin erythema, ulceration of the skin and fins, and hemorrhage and congestion of the visceral organs [4]. Recent studies have shed light on SalHV-3 disease course, tissue distribution, shedding, and sensitivity to a commonly used disinfectant in aquaculture [5,6,7]; however, the virus has yet to be cultured in vitro.

A previous study found that upon experimental bath challenge of juvenile lake trout with tissue homogenates collected and prepared from fish infected with SalHV-3, the virus could first be detected in ocular tissue as early as 9 days post exposure, then spread to multiple visceral organs by day 21 post exposure, but with a clear virus predilection of the eyes, skin, and gill tissues [5]. The median lethal dose of SalHV-3 via bath immersion was determined to be 4.7 × 10^4^ virus copies/mL water [5]. In comparison, viral loads in dermal and ocular tissues reached titers of greater than 10^8^ virus copies per milligram [5]. Gross disease signs observed in experimentally challenged fish (e.g., ocular changes, ulceration of the skin and fins) were consistent with disease signs reported during natural EED epizootics [4,5].

Another study demonstrated that SalHV-3-infected, two-year-old lake trout shed the virus for at least 9 weeks post-exposure and at relatively high loads (i.e., up to 10^9^ virus copies per fish per hour), and the virus could be detected in, and shed from, surviving fish for at least a year [7]. Most remarkable was the ability of some fish to survive an infection dose that killed the majority of other fish in the same tanks. This observation raised several questions, such as: (1) can SalHV-3 persist in fish without causing overt disease, and if so, for how long?; (2) can surviving fish continue to shed the virus under culture conditions with its associated stressor episodes?; and (3) is SalHV-3 and its associated disease restricted to juvenile lake trout, and if not, what kind of tissue distribution and clinical signs can be associated with such an infection in older fish? Unfortunately, none of the previously published field or laboratory studies addressed these questions, creating a gap in knowledge that is vital to designing effective strategies for the control of EED under hatchery conditions and beyond.

In the current study, a group of adult lake trout that had survived a natural SalHV-3 outbreak associated with >25% cumulative mortality [4] were analyzed to determine if: (a) clinical EED could be induced in these fish nearly a decade following clinical recovery; (b) as in infected juvenile lake trout, the virus would be most associated with ocular and epithelial tissues; and (c) virus shedding into the surrounding water would occur at the same intensity as seen with juvenile lake trout. To answer these questions, fish were subjected to either repeated or intermittent handling stress that mimicked what is routinely experienced by captive hatchery broodstock. The ability to maintain recovered SalHV-3-infected fish for nearly a decade provided an extraordinarily rare opportunity to better understand the potential of SalHV-3 to impact lake trout populations indigenous to the Great Lakes of North America.

## 2. Materials and Methods

### 2.1. Fish and Husbandry

This study was conducted in ten-year-old lake trout (average 59.8 cm total length, 2.15 kg) that survived a naturally occurring EED outbreak in 2012 [4], were transferred from the Michigan Department of Natural Resources (MI-DNR) to the Michigan State University—Research Containment Facility (East Lansing, Michigan)—in 2013, and were subsequently reared under quarantine conditions until 2021 in accordance with the Michigan State University Institutional Animal Care and Use Committee guidelines and approval (MSU IACUC; Protocol numbers 07/12-124-99, 11/14-201-00, 11/17-197-00). During this study, fish were housed in two 680 L fiberglass aquaria receiving continuous, ultraviolet-treated, oxygenated well water (12 ± 2 °C), fed AquaMax Sport Fish 600 (Nestlé Purina, St. Louis, MO, USA) to satiation, and tanks cleaned via siphon as needed to remove detritus. The study was conducted in accordance with MSU IACUC guidelines and approval (Protocol number 202000030).

### 2.2. Fish Handling and Sample Collection

#### 2.2.1. Water Sample Collection to Assess Virus Shedding

Prior to the start of the present study, fish were divided into two continuous flow-through 680 L tanks: nine fish were placed in Tank 1 (i.e., Group 1) and 10 fish in Tank 2 (i.e., Group 2). To determine if there was any potential virus shedding occurring, on sampling days, water flow to both tanks was turned off for one hour, while supplemental aeration was maintained and fish monitored continuously. Following fish being held for one hour in the two static holding tanks, water (~40 mL) was collected from each tank for SalHV-3 detection and quantification [7]. Additional water samples were collected each week from the holding and/or sedation tank after all fish had been sedated (for non-lethal sampling) and transferred back to their primary tanks. The collected water samples are summarized in Section 3.5.

#### 2.2.2. Handling and PIT-Tagging

On day 0, after the one-hour holding period to assess virus shedding, and to mimic the handling stress experienced by captive broodstock in hatcheries (e.g., during gamete collection), fish were transferred via net to a 378 L holding tank containing aerated tank water, and the main tank water supply was turned back on. Each fish was sedated with 0.1 g/L tricaine methanesulfonate (MS-222; Syndel, Ferndale, WA, USA) buffered with 0.2 g/L sodium bicarbonate (Church & Dwight Co. Inc., Ewing, NJ, USA). Once sedated, the fish were injected with a 9 mm passive integrated transponder (PIT) tag (Biomark, Boise, ID, USA) in the dorsal musculature following industry standard practices for salmonid broodstocks so that each fish could be individually tracked throughout the study. Samples were then taken non-lethally as detailed below, after which the fish were returned to their flow-through tanks and monitored for recovery. Both groups of fish were likewise handled on days 0 and 49. On Days 7, 14, 21, 28, 35, and 42, Group 1 fish were handled in this manner (repeated handling stress) while Group 2 fish were not (intermittent handling stress). On Day 49, Group 2 fish were handled exactly as on day 0. In contrast, after being netted out of their holding tank and prior to tissue sample collection, Group 1 fish were euthanized with an overdose of MS-222 (0.25 g/L) buffered with sodium bicarbonate (0.5 g/L).

#### 2.2.3. Non-Lethal Tissue Sample Collection

Non-lethal tissue samples were collected from all fish in both groups on Days 0 and 49. Additionally, non-lethal samples were also collected from Group 1 fish on Days 7, 14, 21, 28, 35, and 42. Once each fish was sedated, non-lethal samples were collected as follows:

1. Mucus—approximately 100 µL of mucus per fish was collected from along the left lateral line and around the base of the left pectoral and pelvic fins, using a 1 mL slip-tip syringe barrel (Becton, Dickinson and Company, Franklin Lakes, NJ, USA).
2. Blood—less than 2 mL of whole blood was collected from each fish (maximum of 0.3% body weight), via caudal vessel puncture using a 3 mL luer lock syringe and 20-gauge needle (Becton, Dickinson and Company, Franklin Lakes, NJ, USA). Whole blood was then centrifuged (5000 rpm, 10 min, 4 °C), after which serum was collected.
3. Fin clip—approximately 10 mg tissue was collected using sterile scissors from the left or right pectoral fin.

Following collection, all mucus, serum, and fin tissue samples were immediately frozen at −80 °C for subsequent SalHV-3 testing as detailed below.

#### 2.2.4. Lethal Tissue Sample Collection

**Figure 1:**
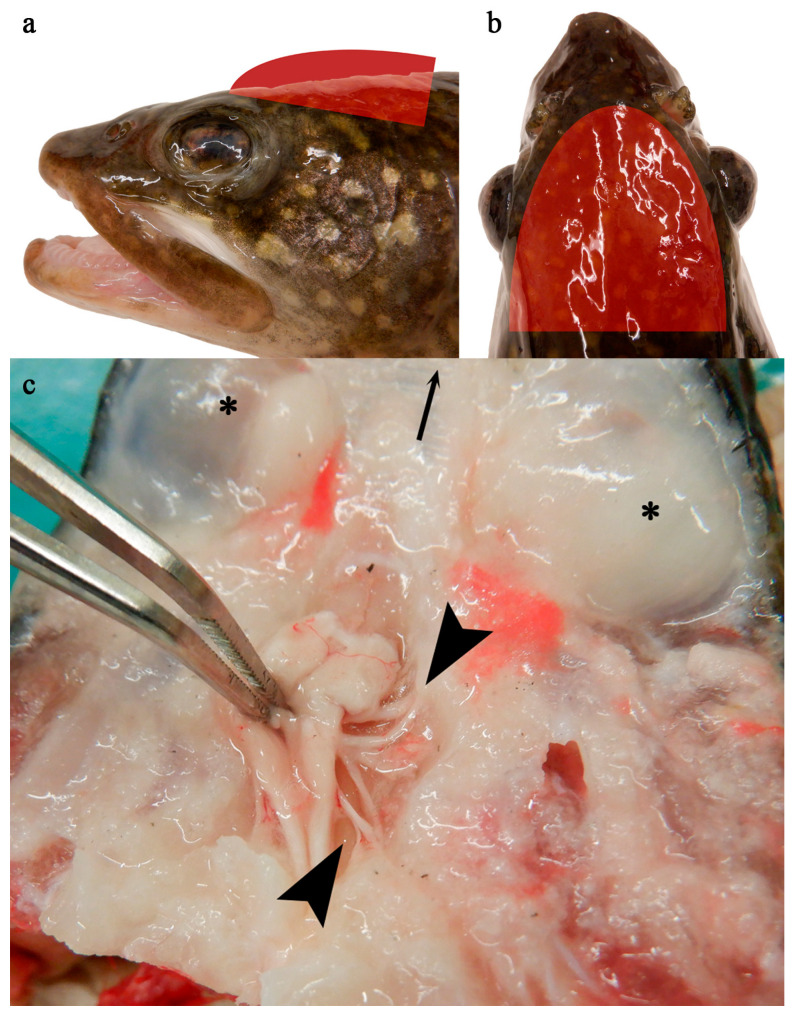
Group 1 fish, apparently normal nervous tissue. Red semi-ellipses indicate approximate level at which transection was made, along a dorsal plane immediately dorsal to the level of the eyes, to expose the brain and associated nervous tissues (**a**,**b**). Brain (held by forceps) and cranial nerve bundle (between arrow heads); dorsal aspect of ocular globes indicated with an *; arrow at top of image indicates rostral direction (**c**).

To assay for SalHV-3 DNA in internal organs following repeated handling stress episodes, Group 1 fish were euthanized on Day 49, and a full clinical examination and necropsy were performed on each. Likewise, tissues from the cerebellum, cerebrum, cornea, cranial nerves (Figure 1), gills, medulla oblongata, olfactory tissue, optic lobe, optic nerve, and gonads were collected from all fish, as were tissues showing additional lesions of concern, and frozen at −80 °C for further analyses.

### 2.3. Detection and Quantification of SalHV-3

#### 2.3.1. DNA Extraction

*Salmovirus salmonidallo3* has yet to be cultured in vitro; thus, molecular methods were utilized. Nucleic acid extractions were performed on collected tissues using the Mag Bind^®^ Blood and Tissue DNA Kit (OMEGA Bio-tek, Inc., Norcross, GA, USA), following the manufacturer’s instructions (saliva protocol for mucus and serum, tissue protocol for all other samples) and with the addition of a filtering step using the E-Z 96^®^ Lysate Clearance Plate (OMEGA Bio-tek, Inc.) [8]. Approximately 10 mg of solid tissue was used for each extraction, and a maximum of 250 µL mucus, serum or swim bladder fluid.

Nucleic acid extractions from water were performed following the Alternative PowerSoil Protocol for Low Bacterial Biomass Fluids using the Qiagen DNeasy^®^ PowerLyzer^®^ PowerSoil^®^ Kit (Qiagen, Hilden, Germany) with minor modifications that also included mechanical disruption via bead-beating [7]. Frozen water samples were thawed to room temperature and vortexed briefly. The bead solution (500 µL), phenol:chloroform (200 µL; isoamyl alcohol; AMRESCO, Solon, OH, USA), and the C1 solution (60 µL) were added to the supplied bead tubes. 250 µL of the water sample was then added into this mixture, vortexed briefly, loaded into the bead beater (Mini-Beadbeater-16; Biospec Inc., Bartlesville, OK, USA) and run on high for 30 s twice with a 20 s rest period between the two bead beating cycles. The mixture was then centrifuged at 10,000× *g* for 1 min at 4 °C. The supernatant was collected and transferred into a new tube provided in the kit, and 1 µL of RNase A was added, followed by 100 µL C2 solution and 100 µL C3 solution. Tubes were then vortexed and incubated at 4 °C for 5 min. Samples were centrifuged for 1 min at 10,000× *g*, and the supernatant was transferred to a new tube. 650 µL C4 solution and 650 µL 100% ethanol were then added to each sample. The remaining steps followed the manufacturer’s instructions, with the addition of the C6 solution being heated to 60 °C before being used to elute the DNA.

Extracted DNA from both protocols was quantified using a Qubit™ fluorometer (Invitrogen, Eugene, OR, USA), and samples were diluted with sterile DNase-free water to no greater than 12.5 ng/µL qPCR template DNA (50 ng per qPCR reaction).

#### 2.3.2. Quantitative PCR Analysis

All qPCR reactions were carried out in a QuantStudio 3 real-time PCR system (ThermoFisher Scientific, Waltham, MA, USA), and were performed using the primers as described by Glenney et al. [8] and as detailed in Shavalier et al. [5]. Each 20 µL reaction contained 10 µL of SYBR^®^ Select Master Mix, 2 µL of nuclease-free water (Promega), 1.0 µM of each primer, and template containing a maximum of 50 ng total DNA. The qPCR cycling parameters consisted of 50 °C for 2 min; 95 °C for 10 min; and 40 cycles of 95 °C for 15 s, and 60 °C for 60 s. Previously confirmed SalHV-3-positive tissue homogenate served as a positive extraction control (PEC), and sample diluent was used as a negative extraction control (NEC). SalHV-3-positive purified DNA and nuclease-free water served as the positive reaction control (PRC) and negative reaction control (NRC), respectively. Samples were considered SalHV-3-positive if amplification occurred within 35 amplification cycles as determined with the QuantStudio 3 accompanying software (ThermoFisher Connect platform, Diomni™ Design and Analysis (RUO) 3.1.0) and the manufacturer’s default settings. Positive control standards for quantification were produced using known positive skin samples following the method outlined by Glenney et al. [8].

Viral loads in tissues and shedding rates (viral copies per fish per hour) were calculated using the resulting reaction copy number calculated by the QuantStudio 3 accompanying software, sample volume or weight, and sample period length.

## 3. Results

### 3.1. Clinical Observations

Over the nearly 9 years that these naturally SalHV-3-infected lake trout were held in quarantine, they experienced one additional and confirmed outbreak of EED in one of three holding tanks in use at the time. That particular tank experienced >99% mortality, and SalHV-3 was detected in 100% of fish tested from the afflicted lot (*n* = 20/20 gills) [4]. This event occurred one year after the initial outbreak in the hatchery, and ~six months after being moved to quarantine (i.e., in the fall of 2013). The surviving fish grew to adulthood in captivity. With the combination of this disease outbreak, occasional mortality or euthanasia of moribund fish, and periodic culling of select individuals over the years for virus screening and experimental stock production, 19 fish were available for the experiment conducted herein.

**Figure 2:**
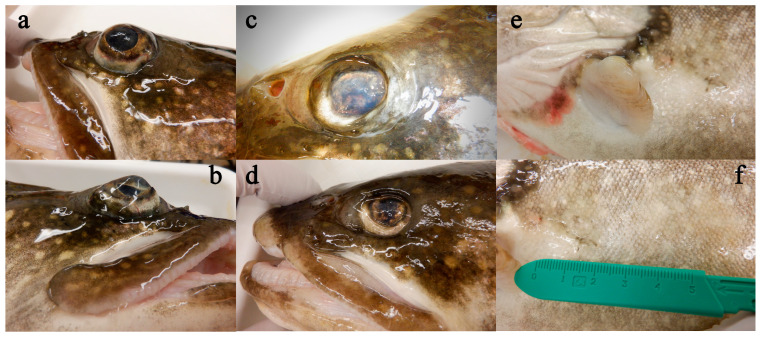
Representative external gross findings in Group 1 fish include exophthalmia (**a**,**b**), enophthalmia (**c**,**d**), corneal opacity (**c**), corneal scarring/thickening (**d**), and chronic fin and skin erosions (**e**,**f**).

No mortality occurred throughout the present experiment in either the repeated handling group or the intermittent handling group. Likewise, neither group of fish showed observable alterations in behavior or appetite throughout the seven weeks of the study; however, some clinical signs associated with EED were observed during the postmortem clinical examination. Fish in Group 1 showed fin and skin erosion ranging from mild to severe, as well as various ocular changes, including exophthalmia (Figure 2a,b), enophthalmia (Figure 2c,d), corneal opacity (Figure 2c) and corneal scarring/thickening (Figure 2d). Although ocular changes were seen in all nine fish, chronic skin and fin changes (Figure 2e,f) appeared more pronounced in the fish with detectable levels of SalHV-3 (see below) than in those without. Internally, observed gross signs of disease included focal hemorrhage within the gonads, renal congestion, swelling of the spleen, hepatic pallor, and mild congestion of the gastric and intestinal vessels. No obvious trends were noted when comparing internal exams of fish from which SalHV-3 was detected and those that were below the detection level.

### 3.2. Non-Lethal Samples—Group 1, Repeated Handling

A total of 216 tissue samples were collected from nine EED-surviving lake trout that were sampled weekly over seven weeks. Across all rounds of nucleic acid extraction and qPCR, negative controls (NEC and NRC) yielded no amplification. Positive extraction controls from previously positive skin homogenates yielded amplification within the expected range (e.g., Ct values 24–31; mean 3.2 × 10^4^ copies per reaction), as did positive reaction control standards at 10-fold dilutions (Ct values ranging from 17–33). Five of the nine fish had at least one tissue where SalHV-3 was detected via qPCR (Table 1 and Table 2), with three having detectable virus DNA at Day 0. Although three fish had virus DNA detections across multiple days, only one fish had detectable SalHV-3 on every sampling day (Table 1 and Table 2). Of the 71 fin samples tested, 18 had detectable SalHV-3 (from *n* = 5 fish), with viral loads ranging from 3.88 × 10^2^ to 8.01 × 10^6^ virus copies per mg of tissue (mean of 1.14 × 10^6^; median of 1.22 × 10^5^). In comparison, SalHV-3 was detected in 13 mucus samples (from *n* = 5 fish) at loads ranging from 6.10 × 10^3^ to 1.01 × 10^7^ virus copies per mL tissue (mean of 2.13 × 10^6^; median of 8.41 × 10^4^). SalHV-3 was detected in a single serum sample throughout the study (1.78 × 10^4^ virus copies/mL tissue; Day 14). Collectively, qPCR analyses revealed a range of patterns in individual fish, whereby some fish were consistently positive or negative throughout, and others varied over the course of repeat sampling (Table 1 and Table 2).

### 3.3. Non-Lethal Samples—Group 2, Intermittent Handling

A total of 60 samples were collected from 10 fish in Group 2, seven weeks apart. SalHV-3 was not detected in any serum samples from intermittently sampled fish. However, there was a total of 11 SalHV-3 detections in fin or mucus samples from 5 fish across both sampling days (Table 1 and Table 2). Virus loads in the fin tissues (*n* = 5) ranged from 1.95 × 10^2^ to 5.31 × 10^5^ viral copies per mg tissue (mean of 1.38 × 10^5^; median of 1.08 × 10^5^). Virus loads in the mucus samples (*n* = 6) ranged from 1.36 × 10^4^ to 4.50 × 10^6^ viral copies per mL tissue (mean of 9.26 × 10^5^; median of 8.90 × 10^4^). Four of the five fish had detectible SalHV-3 on both sampling days, although not necessarily in the same tissues (e.g., SalHV-3 was detected in two fish from fin tissues on day 0 but mucus on day 49). One fish had detectible SalHV-3 (mucus) on the second sampling day only.

**Table 1:** Presence (virus copies/mg tissue) or absence (-) of SalHV-3 DNA as detected by qPCR in non-lethally collected fin tissues. Blank spaces represent tissues not collected.

	Fish #	Study Day							
		0	7	14	21	28	35	42	49
Group 1—Repeated Handling	1	-	3.88 × 10^2^	-	-	-	-	-	-
	2	1.09 × 10^6^	1.43 × 10^6^	3.99 × 10^4^	2.13 × 10^4^	1.15 × 10^5^	2.33 × 10^4^	6.21 × 10^4^	3.44 × 10^5^
	3	-	-	-	-	-	-	-	-
	4	-	-	-	5.87 × 10^2^	-	-	-	-
	5	-	-	-	-	-	-	-	-
	6	-	-	-	-	-	-	-	-
	7	3.91 × 10^6^	9.41 × 10^5^	8.01 × 10^6^	2.53 × 10^6^	-	5.48 × 10^5^	1.00 × 10^5^	1.28 × 10^6^
	8	-	-	-	-	-	-	-	-
	9	5.43 × 10^2^	-	-	-	-	-	-	-
Group 2—Intermittent Handling	10	-							-
	11	1.95 × 10^2^							-
	12	-							-
	13	-							-
	14	-							-
	15	5.31 × 10^5^							-
	16	1.22 × 10^5^							3.64 × 10^4^
	17	-							-
	18	-							1.15 × 10^3^
	19	-							-

**Table 2:** Presence (copies/mL tissue) or absence (-) of SalHV-3 DNA as detected by qPCR in non-lethally collected mucus tissues. Blank spaces represent tissues not collected.

	Fish #	Study Day							
		0	7	14	21	28	35	42	49
Group 1—Repeated Handling	1	-	-	-	-	-	-	-	-
	2	-	-	6.61 × 10^3^	8.90 × 10^4^	-	2.89 × 10^4^	-	-
	3	-	-	-	-	-	-	-	-
	4	-	1.01 × 10^7^	6.13 × 10^6^	1.07 × 10^6^	-	5.40 × 10^4^	6.13 × 10^3^	9.90 × 10^6^
	5	-	-	-	-	-	-	-	-
	6	-	-	-	-	-	-	-	-
	7	-	-	-	8.41 × 10^4^	8.23 × 10^3^	-	-	-
	8	-	-	-	-	-	-	-	1.53 × 10^5^
	9	-	-	3.69 × 10^4^	-	-	-	-	-
Group 2—Intermittent Handling	10	-							1.05 × 10^5^
	11	-							5.15 × 10^5^
	12	-							-
	13	-							-
	14	-							-
	15	-							4.06 × 10^5^
	16	-							2.04 × 10^4^
	17	-							-
	18	1.36 ×10^4^							4.50 × 10^6^
	19	-							-

### 3.4. Lethal Samples—Group 1, Repeated Handling

Among the samples collected at the end of the study from the euthanized fish in Group 1, SalHV-3 DNA was not detected in any cerebellum, cerebrum, gill, or reproductive tissues via qPCR (Table 3). SalHV-3-positive tissues included 3/9 corneas (viral load ranging from 1.34 × 10^2^ to 6.50 × 10^5^ copies per mg tissue; mean of 2.37 × 10^5^; median of 6.04 × 10^4^), 4/9 cranial nerves (viral load ranging from 2.10 × 10^2^ to 3.38 × 10^3^ viral copies per mg tissue; mean of 1.01 × 10^3^; median of 2.30 × 10^2^), 1/9 medulla oblongata (1.14 × 10^2^ viral copies per mg tissue), 1/8 olfactory tissues (1.49 × 10^2^ viral copies per mg tissue), 1/9 optic lobes (2.69 × 10^3^ viral copies per mg tissue), 1/9 optic nerves (2.94 × 10^1^ viral copies per mg tissue), and 1/1 swim bladder fluid (9.41 × 10^4^ viral copies per mL). Positive tissues were detected in six of the nine euthanized fish, with five of the six having at least two positive tissues. Among the six fish with detectable SalHV-3 DNA, four yielded both positive nervous/ocular (i.e., one or more of the following tissues: cranial nerve, medulla oblongata, olfactory, optic lobe, optic nerve, cornea) and external tissues (i.e., fin or mucus), with the remainder having detectable SalHV-3 DNA in only nervous (*n* = 1 fish) or external (*n* = 1 fish) tissues (Table 3).

### 3.5. SalHV-3 Shedding

A total of eight MS-222 water samples were collected following sedation of the Group 1 (repeated handling) fish on days 0, 7, 14, 21, 28, and 42, and the Group 2 (intermittent handling) fish on days 0 and 49. Of these, one sample had detectable SalHV-3 (Day 7, Group 1 repeated handling) at an estimated concentration of 2.36 × 10^3^ copies/mL water. A total of 14 water samples were collected from the two fish tanks following 1 h of static holding (both Group 1 and Group 2 fish on days 0, 7, 14, 21, 28, 42, and 49). Of these, SalHV-3 was detected in one water sample (Day 7, Group 2 intermittent handling) at a concentration of 2.04 × 10^4^ copies/mL water.

**Table 3:** Number of SalHV-3-positive tissue samples by fish throughout the study.

	Tissue Type															
		Non-Lethal				Lethal										
	Fish #	Fin	Mucus	Serum			Cerebellum	Cerebrum	Cranial Nerve	Medulla Oblongata	Olfactory	Optic lobe	Optic Nerve	Cornea	Gill	Gonads
Group 1—Repeated Handling	1	0/8	0/8	0/8			0/1	0/1	0/1	0/1	0/1	0/1	0/1	0/1	0/1	0/1
	2	***8/8***	***3/8***	0/8			0/1	0/1	***1/1***	0/1	0/1	***1/1***	***1/1***	***1/1***	0/1	0/1
	3	0/8	0/8	0/8			0/1	0/1	***1/1***	0/1	***1/1***	0/1	0/1	0/1	0/1	0/1
	4	***1/8***	***6/8***	***1/8***			0/1	0/1	0/1	0/1	-	0/1	0/1	0/1	0/1	0/1
	5	0/8	0/8	0/8			0/1	0/1	0/1	0/1	0/1	0/1	0/1	0/1	0/1	0/1
	6	0/8	0/8	0/8			0/1	0/1	0/1	0/1	0/1	0/1	0/1	0/1	0/1	0/1
	7	***7/8***	***2/8***	0/8			0/1	0/1	***1/1***	0/1	0/1	0/1	0/1	***1/1***	0/1	0/1
	8	0/8	***1/8***	0/8			0/1	0/1	***1/1***	***1/1***	0/1	0/1	0/1	0/1	0/1	0/1
	9	***1/8***	***1/8***	0/8			0/1	0/1	0/1	0/1	0/1	0/1	0/1	***1/1***	0/1	0/1
Group 2—Intermittent Handling	10	0/2	***1/2***	0/2			-	-	-	-	-	-	-	-	-	-
	11	***1/2***	***1/2***	0/2			-	-	-	-	-	-	-	-	-	-
	12	0/2	0/2	0/2			-	-	-	-	-	-	-	-	-	-
	13	0/2	0/2	0/2			-	-	-	-	-	-	-	-	-	-
	14	0/2	0/2	0/2			-	-	-	-	-	-	-	-	-	-
	15	***1/2***	***1/2***	0/2			-	-	-	-	-	-	-	-	-	-
	16	***2/2***	***1/2***	0/2			-	-	-	-	-	-	-	-	-	-
	17	0/2	0/2	0/2			-	-	-	-	-	-	-	-	-	-
	18	***1/2***	***2/2***	0/2			-	-	-	-	-	-	-	-	-	-
	19	0/2	0/2	0/2			-	-	-	-	-	-	-	-	-	-

## 4. Discussion

Although findings from the present study are based upon a relatively small number of EED-recovered fish (*n* = 19), their maintenance for nearly a decade allowed several heretofore unknown aspects of SalHV-3 pathogenesis to be uncovered. First, *Salmovirus salmonidallo3* DNA was detected in adult lake trout 8 years and 7 months following a natural disease outbreak. Gross signs of disease as detailed above were consistent with what is typically seen in lake trout that have previously experienced an active SalHV-3 infection, either natural or experimentally induced (chronic ocular, skin, and fin changes) [4,5]. Because this group of fish was held in quarantine for nearly a decade, the detected virus in these fish definitively originated from the natural outbreak. Whether SalHV-3 was present as a persistent infection in surviving lake trout or went through latency and became reactivated due to the handling stress is currently unknown.

Latency or viral persistence has been demonstrated in several other alloherpesviruses, including the cyprinid herpesviruses (*Cyvirus cyprinidallo1*, CyHV-1; *Cyvirus cyprinidallo2*, CyHV-2; *Cyvirus cyprinidallo3*, CyHV-3), and *Ictavirus ictalluridallo1* (IcHV-1) [9,10,11,12], and a recent review (Quijano Cardé and Soto, 2024) highlighted the current knowledge of *Alloherpesviridae* in this context [13]. Eide et al. [14] demonstrated latency and viral reactivation in koi (*Cyprinus carpio*) previously exposed to koi herpesvirus (CyHV-3), with the time interval between disease outbreak and virus reactivation reported as “several years”. Baumer et al. [11] also examined latency in CyHV-3, demonstrating viral shedding in surviving fish up to 21 months post-exposure. Subclinical infection with *Cyvirus cyprinidallo2* in one-year-old gibel carp (*Carassius gibelio*) was described by Wei et al. [15], who were also able to reactivate the production of infectious virus. Latency and reactivation has been of much interest with *Ictavirus ictalluridallo1* as well; however, while virus DNA has occurred in various groups of subclinical fish, reactivation of clinical disease has proven challenging to achieve in a laboratory setting [16]. Results from the present study likewise indicate that an alloherpesvirus of lake trout (Family *Salmonidae*), SalHV-3, is capable of generating persistent and/or latent infections of a substantial duration in surviving fish, in this case at least eight and a half years after a naturally occurring EED hatchery outbreak [4].

In a similar context, SalHV-3 DNA was detected in holding tank water during this study, suggesting that eight and a half years after surviving an EED outbreak, some LAT remain capable of shedding the virus and thereby represent a potential long-term reservoir of infection. Although knowledge on shedding dynamics of other alloherpesviruses is incomplete, viral shedding and duration have indeed been investigated in several instances. For example, it has been reported (via analysis of mucosal swabs and in vivo cohabitation experiments) that CyHV-3 can be shed for up to 57 days in experimentally exposed, subclinically affected fish [17] and up to 34 days post-challenge in a separate study [18]. In both instances, longer viral shedding was associated with colder water temperatures and milder clinical disease. Kancharla and Hanson [19] showed that *Ictavirus ictalluridallo1* shedding peaks around 4 days post-infection and continues for at least 12 days. In the current study, the shedding of SalHV-3 was seemingly intermittent and, when detected, at loads estimated to be ~10^4^ virus copies/mL water/hour. Although lower than the SalHV-3 shedding rates that were detected during the peak of an experimentally induced EED outbreak in juvenile lake trout [7], this virus concentration is near to the estimated median lethal dose via immersion as determined in our previous studies [5]. In the present study, SalHV-3 was detected in a subset of water samples despite a relatively short fish holding time. It is possible that increasing the fish holding duration from one hour to eight hours [7] and incorporating a filtration/ultracentrifugation step [20,21] would have revealed a higher or more consistent virus shedding rate in these EED-surviving fish. Regardless, these findings provide further evidence that EED-recovered adult lake trout constitute a potential SalHV-3 transmission risk for an extended period of time.

Virus shedding via the reproductive fluids (i.e., ovarian fluid, milt) is also of concern for salmonid herpesviruses. For example, Wolf et al. [22] identified *Salmovirus salmonidallo1* in the ovarian fluid of rainbow trout (*Oncorhynchus mykiss*); likewise, *Salmovirus salmonidallo2* has been detected in reproductive fluids, but details on the duration of shedding from infected fish via this mode are unknown [23]. SalHV-3 DNA has also been detected in the ovarian fluid of lake trout on multiple prior occasions [8,24]. Interestingly, SalHV-3 was not detected in the reproductive tissues of the fish in this current study. As these fish were not yet ripe (i.e., ready to spawn) at the time of sample collection, viral presence in gonadal tissues and fluids may relate to spawning status and warrants further study.

The present study was designed to mimic the handling and associated stress hatchery broodstock would experience during routine management procedures (i.e., transfer via netting, holding in a confined space for a period of time, anesthesia) and determine if repeated (Group 1) or intermittent (Group 2) handling affected reactivation of SalHV-3 in terms of virus loads in adult LAT that survived a previous, natural EED outbreak. In so doing, several notable observations were made. First, the mean viral load was 2.3–8.3-fold higher in the mucus and fins, respectively, of fish that were subjected to repeated handling (Group 1) compared to those that were only intermittently handled (Group 2). Second, and although only in one fish, SalHV-3 was exclusively detected in the serum of fish within the repeated handling group. The total percentage of positive fish identified by fin clip was nearly identical between the two treatment groups (24% versus 25%), whereas the percentage of SalHV-3 mucus samples was interestingly higher in the intermittently handled (Group 2) fish. Thus, it is possible that in some cases, a single handling stressor may result in just as much risk of virus reactivation as repeated handling stress. Notably, when comparing the mucus samples collected on day zero to those taken on day 49, there was an increase in the number of fish in both groups with detectable levels of SalHV-3, with the repeat sampling group going from 0/9 on day zero to 2/9 on day 49 and the intermittent group going from 1/10 on day zero to 5/10 on day 49.

Studies on other alloherpesviruses have found that stressful events favor reactivation of productive infections [25]. A study on persistently infected common carp demonstrated that netting fish was enough to reactivate CyHV-3 (measured by an increase in viral load in gill swabs) [26]. Captive broodstock can also experience unusual levels of stress during times of intense management (e.g., handling of the fish for movement, vaccination, PIT tagging, etc. [27]), as well as during spawning seasons, leading to an increased risk for herpesvirus reactivation in persistently or latently infected fish, and increased risk of virus transmission due to increased fish density and natural seasonal immune system changes [28]. Identification of infected broodstock, coupled with management planning to limit or reduce stress experienced by the fish, can help to prevent additional spread of the virus.

Results from this study also illustrate that SalHV-3 infects more tissue types than previously recognized. It is well documented that many herpesviruses target different tissues or cell types during an acute infection versus those they infect to establish latency [13,29]. Although previous studies have identified a tropism for epithelia of the skin, fin, gills, and cornea during acute SalHV-3 infections and EED events, and thus are commonly utilized for screening and diagnostic purposes [4,5], prior to this study, the potential targets during long-term SalHV-3 infections were unknown. Other herpesviruses, including many of the human herpesviruses, as well as *Cyvirus cyprinidallo1* and *Cyvirus cyprinidallo2* [25,30,31], are known to develop latency in various nervous tissues, and Tolo et al. reported that CyHV-3 DNA can commonly be detected in brain tissues despite no evidence of viral replication [32]. However, the specific neurotropism of SalHV-3 was previously unexplored. The present study expanded upon previous work that identified SalHV-3 DNA in undifferentiated “brain” tissue [5] by taking advantage of larger adult fish, which allowed for the identification, dissection, and testing of individual nervous tissues. Herein, SalHV-3 DNA was detected in the cranial nerves (including the optic nerve), the medulla oblongata, the optic lobes, and the olfactory tissues collected from inside the nares. Based on trends seen in other herpesviruses, these findings may suggest a neurotropic target for latent SalHV-3 infections pending further investigation. Interestingly, ocular tissues (i.e., cornea) have been identified as an early target during SalHV-3 infections [5], and gross ulcers around the nares are commonly reported during active disease events [4]. Thus, the presence of SalHV-3 DNA in the nervous tissues associated with these areas (olfactory tissue, optic nerve, optic lobe) as identified in this study, points toward these tissues as likely virus targets in fish during advanced stages of infection. Although additional research is needed to determine if these fish were truly latently infected or not, this study highlights the importance of targeting nervous tissues, specifically those within the head, for further investigations into SalHV-3 latency development.

In conclusion, this study demonstrates that SalHV-3 can persist in adult lake trout that survived a natural EED outbreak well beyond previously identified carrier time frames that have been determined thus far for other alloherpesviruses. As these infected fish have also been demonstrated to shed the virus, long-term survivor fish likely represent an important reservoir and virus transmission risk in hatchery environments, and appropriate control and containment measures must be put in place to prevent the spread of SalHV-3 virus to naïve fish populations.

## Data Availability

Data is contained within the article.

## Abbreviations

The following abbreviations are used in this manuscript:

SalHV-3	*Salmovirus salmonidallo3*
CyHV-3	*Cyvirus cyprinidallo3*
EED	Epizootic Epitheliotropic Disease
PIT	Passive Integrated Transponder
MS-222	Tricaine Methanesulfonate
qPCR	Quantitative PCR

## References

[1] Walker P.J., Siddell S.G., Lefkowitz E.J., Mushegian A.R., Adriaenssens E.M., Alfenas-Zerbini P., Dempsey D.M., Dutilh B.E., García M.L., Hendrickson R.C. (2022). Recent changes to virus taxonomy ratified by the International Committee on Taxonomy of Viruses (2022). Arch. Virol..

[2] Bradley T.M., Medina D.J., Chang P.W., McClain J. (1989). Epizootic epitheliotrophic disease of lake trout (*Salvelinus namaycush*): History and viral etiology. Dis. Aquat. Org..

[3] Bradley T., Newcomer C., Maxwell K. (1988). Epitheliocystis associated with massive mortalities of cultured lake trout *Saivelinus namaycush*. Dis. Aquat. Org..

[4] Faisal M., Loch T.P., Shavalier M., VanDeuren M.G., Standish I., Winters A., Glenney G., Aho J., Wolgamood M., VanAmberg J. (2019). Resurgence of Salmonid Herpesvirus-3 Infection (Epizootic Epitheliotropic Disease) in Hatchery-Propagated Lake Trout in Michigan. J. Aquat. Anim. Health.

[5] Shavalier M., Faisal M., Loch T.P., Fitzgerald S.D., Thaiwong T., Kiupel M. (2020). Disease Progression in Lake Trout (*Salvelinus namaycush*) Experimentally Infected With Epizootic Epitheliotropic Disease Virus (Salmonid Herpesvirus-3). Vet. Pathol..

[6] Purbayu M.A., Shavalier M.A., Faisal M., Loch T.P. (2021). Experimental Evidence of Epizootic Epitheliotropic Disease Virus (Salmoid Herpesvirus-3, *Alloherpesviridae*) Transmission via Contaminated Fomites and Subsequent Prevention Using a Disinfectant. Pathogens.

[7] Faisal M., Purbayu M., Shavalier M.A., Marsh T.L., Loch T.P. (2019). Shedding of the Salmonid Herpesvirus-3 by Infected Lake Trout (*Salvelinus namaycush*). Viruses.

[8] Glenney G.W., Barbash P.A., Coll J.A. (2016). A Quantitative Polymerase Chain Reaction Assay for the Detection and Quantification of Epizootic Epitheliotropic Disease Virus (EEDV; Salmonid Herpesvirus 3). J. Aquat. Anim. Health.

[9] Wei C., Iida H., Chuah Q., Tanaka M., Kato G., Sano M. (2019). Persistence of cyprinid herpesvirus 2 in asymptomatic goldfish *Carassius auratus* (L.) that survived an experimental infection. J. Fish Dis..

[10] Lepa A., Siwicki A.K. (2013). Fish herpesvirus diseases: A short review of current knowledge. Acta Vet. Brno.

[11] Baumer A., Fabian M., Wilkens M.R., Steinhagen D., Runge M. (2013). Epidemiology of cyprinid herpesvirus-3 infection in latently infected carp from aquaculture. Dis. Aquat. Org..

[12] Davison A.J., Eberle R., Ehlers B., Hayward G.S., McGeoch D.J., Minson A.C., Pellett P.E., Roizman B., Studdert M.J., Thiry E. (2009). The order Herpesvirales. Arch. Virol..

[13] Quijano Cardé E.M., Soto E. (2024). A review of latency in the Alloherpesviridae family. J. Fish Dis..

[14] Eide K.E., Miller-Morgan T., Heidel J.R., Kent M.L., Bildfell R.J., LaPatra S., Watson G., Jin L. (2011). Investigation of koi herpesvirus latency in koi. J. Virol..

[15] Wei C., Xu C., Sun Y., Li J., Sano M., Li Q. (2023). Investigation of the latency of *Cyprinid herpesvirus* 2 in apparently healthy farmed gibel carp, *Carassius auratus gibelio*. Aquaculture.

[16] Hanson L., Dishon A., Kotler M. (2011). Herpesviruses that infect fish. Viruses.

[17] Cano I., Mulhearn B., Akter S., Paley R. (2020). Seroconversion and Skin Mucosal Parameters during Koi Herpesvirus Shedding in *Common carp*, *Cyprinus carpio*. Int. J. Mol. Sci..

[18] Yuasa K., Ito T., Sano M. (2008). Effect of water temperature on mortality and virus shedding in carp experimentally infected with koi herpesvirus. Fish Pathol..

[19] Kancharla S.R., Hanson L.A. (1996). Production and shedding of channel catfish virus (CCV) and thymidine kinase negative CCV in immersion exposed channel catfish fingerlings. Dis. Aquat. Org..

[20] Hubbard L.E., Stelzer E.A., Poulson R.L., Kolpin D.W., Szablewski C.M., Givens C.E. (2024). Development of a Large-Volume Concentration Method to Recover Infectious Avian Influenza Virus from the Aquatic Environment. Viruses.

[21] Ip Y.C.A., Chen J., Tan L.Y., Lau C., Chan Y.H., Balasubramaniam R.S., Wong W.Y.J., Ng K., Tan Z.Y.B., Fernandez C.J. (2024). Establishing environmental DNA and RNA protocols for the simultaneous detection of fish viruses from seawater. Environ. DNA.

[22] Wolf K., Darlington R., Taylor W., Quimby M., Nagabayashi T. (1978). Herpesvirus salmonis: Characterization of a new pathogen of rainbow trout. J. Virol..

[23] Hanson L.A., Doszpoly A., van Beurden S., de Oliveira Viadanna P.H., Waltzek T. (2024). Alloherpesviruses of fish. Aquaculture Virology.

[24] Kurobe T., Marcquenski S., Hedrick R. (2009). PCR assay for improved diagnostics of epitheliotropic disease virus (EEDV) in lake trout *Salvelinus namaycush*. Dis. Aquat. Org..

[25] Grinde B. (2013). Herpesviruses: Latency and reactivation–viral strategies and host response. J. Oral Microbiol..

[26] Bergmann S., Kempter J. (2011). Detection of koi herpesvirus (KHV) after re-activation in persistently infected common carp (*Cyprinus carpio* L.) using non-lethal sampling methods. Bull. Eur. Assoc. Fish Pathol..

[27] Bordeleau X., Hatcher B.G., Denny S., Fast M.D., Whoriskey F.G., Patterson D.A., Crossin G.T. (2018). Consequences of captive breeding: Fitness implications for wild-origin, hatchery-spawned Atlantic salmon kelts upon their return to the wild. Biol. Conserv..

[28] Tort L., Balasch J.C., Mackenzie S. (2003). Fish immune system. A crossroads between innate and adaptive responses. Inmunología.

[29] Cohen J.I. (2020). Herpesvirus latency. J. Clin. Investig..

[30] Sano N., Moriwake M., Hondo R., Sano T. (1993). Herpesvirus cyprini: A search for viral genome in infected fish by infected fish by in situ hybridization. J. Fish Dis..

[31] Chai W., Qi L., Zhang Y., Hong M., Jin L., Li L., Yuan J. (2020). Evaluation of Cyprinid herpesvirus 2 latency and reactivation in *Carassius gibel*. Microorganisms.

[32] Tolo I.E., Bajer P.G., Mor S.K., Phelps N.B. (2023). Disease ecology and host range of Cyprinid herpesvirus 3 (CyHV-3) in CyHV-3 endemic lakes of North America. J. Fish Dis..

